# Obesity and Co-morbid Conditions Are Associated with Specific Neuropsychiatric Symptoms in Mild Cognitive Impairment

**DOI:** 10.3389/fnagi.2017.00164

**Published:** 2017-05-29

**Authors:** Ashley H. Sanderlin, David Todem, Andrea C. Bozoki

**Affiliations:** ^1^Neuroscience Program, Michigan State University, East LansingMI, United States; ^2^Division of Biostatistics, Department of Epidemiology and Biostatistics, Michigan State University, East LansingMI, United States; ^3^Department of Neurology and Ophthalmology, Michigan State University, East LansingMI, United States

**Keywords:** mild cognitive impairment, behavioral symptoms, obesity, Alzheimer’s disease, type 2 diabetes

## Abstract

**Background:** Neuropsychiatric symptoms (NPSs) in MCI, and midlife obesity increase the likelihood of developing Alzheimer’s disease. It is unknown whether obesity or related health conditions modify the risk of NPS or severity of cognitive impairment in MCI.

**Methods:** One hundred and thirteen subjects with MCI were assessed near the time of MCI diagnosis. The sample was divided by BMI and related disorders, type-2 diabetes (T2D) and obstructive sleep apnea (OSA) to measure the relationship of these groups with NPS and severity of MCI. NPSs scores were evaluated based on the Neuropsychiatric Inventory-Questionnaire (NPI-Q) and Geriatric Depression Scale, along with NPI-Q clusters. MCI-severity was estimated based on a composite z-score of neuropsychological tests.

**Results:** Obese and overweight subjects represented 65% of the sample and were on average 7 years younger than normal weight subjects. The presence of obesity, T2D and OSA status modified the prevalence and severity of specific NPI-Q symptom clusters, specifically affective symptoms were more frequent across groups and severe in OB and T2D. Total NPS scores were higher for subjects with T2D and OSA although MCI-severity did not differ across groups.

**Conclusion:** MCI subjects with obesity, T2D and OSA demonstrated a higher susceptibility to psychopathologic changes.

## Introduction

Behavioral changes or neuropsychiatric symptoms are prevalent in mild cognitive impairment (MCI) and are associated with an increased likelihood of conversion to dementia (Ismail et al., 2016). Neuropsychiatric symptoms (NPSs) such as depression, anxiety and apathy are a hallmark of Alzheimer’s disease (AD) (Lyketsos et al., 2002; Geda et al., 2008). As high as 80% of AD patients have at least one symptom on the Neuropsychiatric inventory with affective and apathy symptoms having the highest prevalence (Lyketsos et al., 2002; Zhao et al., 2016). MCI is a transitional state between normal cognition and dementia and the presence of NPS predict the progression of MCI to AD, decreasing the time of progression to dementia by 2.5 fold (Palmer et al., 2007; Teng et al., 2007). In MCI depression is one of the most prevalent symptoms and has been directly related to cognitive decline and the development of dementia (Rosenberg et al., 2012a; Gorska-Ciebiada et al., 2014).

Obesity is a disorder characterized by excess body fat with low energy expenditure. Obesity is a contributor to the metabolic syndrome, and is associated with cognitive deficits along with an increased likelihood of developing dementia when present at midlife (Kandimalla et al., 2016). The prevalence of obesity in the U.S. has nearly tripled over the last 30 years and is highest among middle age and older adults (Flegal et al., 2010). Side effects of chronic obesity include lower global brain volume, a high risk for metabolic syndrome, and premature death (Han et al., 2011). Further, obesity affects cognition (Gustafson, 2006) and often occurs co-morbidly with NPS across age groups (Simon et al., 2006). Additional conditions, consequences of obesity such as type 2 diabetes, sleep apnea and other vascular disorders are also associated with cognitive decline and increased NPSs. Multiple lines of evidence demonstrate a link between midlife obesity and the development of dementia (Gustafson, 2006; Beydoun et al., 2008; Whitmer et al., 2008). However, the relationship between NPS and obese subjects within early MCI has not been studied.

Neuropsychiatric symptoms and obesity have not been measured together to determine their co-morbidity in MCI and interactions with cognition. In the present study, our hypothesis is that in MCI, obesity is associated with higher total NPS scores and a higher prevalence and severity of affective symptoms (depression and anxiety), as well as more extensive cognitive loss as measured by MCI severity. We sought to first identify the prevalence of obesity, obesity-related health conditions, and NPS within MCI, examining their relationship and their effect on the severity of cognitive impairment. We then clustered similar NPS together, and examined the frequency and severity of behavioral clusters across weight groups and BMI-related health conditions.

## Materials and Methods

All study data came from medical records dating between 2004 and 2014 from a tertiary geriatric neurology clinic at Michigan State University serving the mid-Michigan area. Clinical and behavioral data were taken at the time of diagnosis with MCI. This study involved minimal risk to human subjects and a waiver of consent was requested and approved by the Michigan State University Institutional Review Board for pre-existing clinical data related to this study. To provide further information, subjects were recruited for the sole purpose of inclusion in this study and informed written consent was received in accordance with the Michigan State University Institutional Review Board.

### MCI Diagnosis

The diagnosis of MCI was determined according to Petersen’s Criteria (Petersen et al., 1999) by an expert neurologist (A. Bozoki). The diagnostic process included an initial clinical evaluation by the neurologist followed by a neuropsychological assessment battery, MRI (head CT if MRI was contraindicated) and serologic testing for metabolic profile, thyroid function, and vitamin B12 level. The neuropsychological assessment battery [a modified CERAD battery (Moms et al., 1989), described in further detail in Section “MCI Severity”], assessed memory, verbal and visual delayed recall, language, visuospatial and executive functions, was administered to all subjects. Subjects scoring ≥-1.5 standard deviations (SD) below the education and age-adjusted mean in one or more cognitive domains were classified as MCI. The MCI sample represented a heterogeneous population consisting of amnestic MCI, non-amnestic MCI, and multi-domain MCI subtypes.

Inclusion criteria were as follows: subjects were between the ages of 50–95, able to speak, comprehend and read English with at least 8 years of education, and a Mini Mental Status Examination (MMSE) (Folstein et al., 1975) score between 24 and 30. Subjects were excluded if they had a history of a coexisting central nervous system disorder or uncontrolled depression that could account for the cognitive impairment, any uncontrolled or unstable medical condition, and alcohol or substance abuse within the last 2 years. Exclusion criteria were determined based on medical records review. Over the 10-year period there were 667 subjects with neuropsychometric data. Of the 667 subjects examined, 117 were diagnosed with MCI. A total of four subjects were excluded from the study due to a history of major depression (*n* = 3) and stroke (*n* = 1). Our final sample consisted of 113 subjects that met the inclusion criteria.

### BMI Groups

The MCI sample was grouped by traditional BMI criteria: normal weight (NW; BMI 18.5–24.9), overweight (OW; BMI 25–29.9), or obese (OB; BMI ≥ 30). Height (in inches) and weight (in pounds) measurements were taken at the time of clinical diagnosis of MCI. BMI was converted to the unit kg/m^2^ using the follow calculation, [(Weight (lb.)/Height^2^ (in.)) × 703].

### BMI-Related Disorders

A clinical history of BMI-related disorders was recorded in order to account for conditions that may be comorbid with increased weight (Misiak et al., 2012) but pose an independent risk factor for cognitive decline (Li et al., 2011), or have an increased prevalence of NPSs (Steinberg et al., 2014). These included, type-2-diabetes (T2D), hypertension (HTN), hyperlipidemia (HL), gastroesophageal reflux disease (GERD), and obstructive sleep apnea (OSA). The presence or absence of each of these conditions was recorded for each subject at the time of MCI diagnosis. In addition, blood pressure recordings at the time of diagnosis were used to calculate a mean arterial pressure (MAP) value for each subject as a measure of cardiovascular health.

### Neuropsychiatric Symptoms

Neuropsychiatric symptom scores along with mild or moderate prevalence groups were measured using the Neuropsychiatric Inventory Questionnaire (NPI-Q) (Kaufer et al., 2000) and the Geriatric Depression Scale –short form (GDS) (Herrman et al., 1996). The NPI-Q is a validated measure for assessing behavioral disturbances across 12 different domains in a brief caregiver-reported questionnaire (Kaufer et al., 2000). These include; delusions, hallucinations, agitation/aggression, depression/dysphoria, anxiety, elation/euphoria, apathy/indifference, disinhibition, irritability, aberrant motor behavior, sleep and nighttime behavioral changes, and appetite and eating disorders. An informant familiar with the subject reported NPI-Q symptoms, by rating each symptom first for their presence (yes/no), and then severity (range of 1–3) with a total of 36 possible points. Behavioral changes reported on the NPI-Q reflect symptoms present within 1 month of testing. The self-reported 15-point GDS scale was used for further quantification of depressive symptoms. Mild NPS was designated as a total score ≥ 1 and moderate NPS as ≥ 4 for each test. The total score for each test and the prevalence of mild and moderate symptoms were measured across groups.

### NPI-Q Clusters

Neuropsychiatric Inventory-Questionnaire symptoms were grouped into clusters based on a prior research study demonstrating that specific NPI symptoms tend to cluster together when they emerge as part of a dementia (Aalten et al., 2007). Benefits of assessing NPI/NPIQ clusters instead of individual symptoms include both examination of underlying similarities in prevalence, progression of symptoms and biological correlates (Aalten et al., 2005). Thus, in the present study the 12 NPIQ symptoms were grouped into four clusters: Hyperactivity (agitation, disinhibition, irritability, motor disturbances, and euphoria), Psychosis (delusions, hallucinations, night-time behaviors), Apathy (apathy, appetite), and Affective (depression, anxiety). The presence of a symptom cluster required the presence of at least one symptom within each cluster. The cluster severity was the average of the total score (0–3) across each symptom within a cluster for each subject.

### MCI Severity

To determine whether BMI was associated with an increase in MCI severity (MCI-SV), a z-score was computed for each cognitive test in the neuropsychological test battery, then averaged to obtain a mean overall z score for each subject. Included test measures evaluated global cognition [MMSE; Modified Mini Mental Exam (3ME) (Teng and Chui, 1987)], memory [CERAD Word List, immediate/delayed/recognition (Atkinson and Shiffrin, 1971)], language [CERAD 15-item Boston Naming Test (Kaplan et al., 1983); categorical and phonemic verbal fluency (Borkowski et al., 1967)], executive function [Trail Making Test (Tombaugh, 2004); Stroop (Stroop, 1935)], and visuospatial tests [CERAD Constructional Praxis, immediate/delayed (Rosen et al., 1984)].

### Statistical Analyses

The analysis of variance (ANOVA) model was used to compare NPI-Q total score, GDS score, NPI-Q cluster severity and MCI severity scores across BMI groups. These comparisons were further adjusted for age and education using the analysis of covariance (ANCOVA) model. Specific BMI-related disorders that had a high prevalence of obesity were also used as independent variables. A chi-square test of independence was conducted to compare the frequency of NPI-Q clusters across BMI groups and BMI-related disorders. A Fisher’s exact test was used to compare frequencies of NPI-Q clusters between groups when cell sample sizes were small. Statistical analysis was conducted using SPSS software (Hewlett Packard; Palo Alto, CA, United States). A two-sided *p*-value less than 5% (*p* < 0.05) was used for statistical significance.

## Results

A description of the study sample can be found in **Table 1**. Of the 113 MCI subjects included in the study, 110 had available BMI data and roughly 1/3 each were NW, OW, and OB. Overall, the average BMI, mean age and MMSE of the sample was 27.4 kg/m^2^, 74.1 years and 26.5 respectively. Over 90% of the sample was Caucasian with an average educational attainment of 14.6 years. Surprisingly, NW subjects were significantly older than OW and OB (*p* < 0.001) and had higher educational attainment (*p* = 0.05). Overall, 78.6% of subjects had at least one symptom on the NPI-Q and 87.3% had one symptom on the GDS. BMI was positively correlated with NPI-Q score (Pearson’s *r* = 0.225; *p* = 0.04), and a direct comparison of NW and OB groups revealed a significantly higher prevalence of NPI-Q symptoms (Student’s *t* = 2.05; *p* = 0.045, unadjusted). However, there was not an effect of BMI on NPS or cognitive measures in the ANOVA model.

**Table 1:** Demographic, cognitive, and behavioral measures of the MCI sample grouped by BMI.

Characteristic	Entire group	NW	OW	OB	Statistic	
	*N* = 113	*N* = 38	*N* = 39	*N* = 33	X^2^ or *F*	*p*-value
Age (years)	74.3 (0.72)	78.74 (1.00)	72.4 (1.33)	71.2 (1.12)	11.97	<0.001
Female, *n* (%)	53 (47)	20 (53)	20 (51)	11 (33)	3.23	0.20
Education (years)	14.5 (0.31)	15.6 (0.49)	14.2 (0.49)	13.8 (0.64)	2.86	0.06
MMSE	26.5 (0.17)	26.0 (0.24)	26.8 (0.29)	26.7 (0.33)	2.24	0.11
MCI-severity^a^	-0.89 (0.06)	-1.04 (0.09)	-0.80 (0.09)	-0.82 (0.14)	4.11	0.02
NPI-Q score^a^	5.2 (0.56)	4.0 (0.72)	5.4 (1.02)	6.7 (1.27)	0.47	0.63
≥1, *n* (%)	68 (79)	25 (74)	26 (87)	17 (77)	1.72	0.42
≥4, *n* (%)	41 (48)	14 (41)	13 (43)	14 (64)	3.05	0.22
GDS score^a^	3.0 (0.26)	3.0 (0.35)	2.7 (0.35)	3.3 (0.73)	0.63	0.53
≥1, *n* (%)	91 (88)	34 (90)	33 (92)	22 (79)	2.70	0.26
≥4, *n* (%)	32 (31)	14 (37)	9 (25)	8 (29)	1.29	0.53

*MCI, mild cognitive impairment; BMI, body mass index; MCI SV, MCI severity; MMSE, Mini-Mental State Examination; MCI-SV, MCI severity; GDS, Geriatric Depression Scale; NPI-Q, Neuropsychiatric Inventory Questionnaire; NW, normal weight; OW, overweight; OB, obese. Values are presented as mean (standard error) for continuous variables and *n* (%) for categorical variables. The statistic is chi-square test of independence for categorical variables (2 degrees of freedom) and an ANOVA *F*-statistic for continuous variables. Available behavioral data was NPI-Q, *n* = 86 and GDS, *n* = 102. ^a^Adjusted for age and education.*

The frequency of all examined BMI-related disorders are displayed in **Table 2**. There was no difference in HTN, HL, GERD, and MAP across BMI groups. However, a significantly higher proportion of T2D and OSA subjects were OB, thus, T2D and OSA were used as independent variables in further analysis of individual NPI-Q cluster frequency and severity.

**Table 2:** The frequency of BMI-related disorders within BMI groups.

	Entire group	NW	OW	OB	X^2^ or *F*	*p*-value
GERD	14%	16%	13%	27%	0.24	0.89
HP	43%	34%	44%	52%	2.18	0.34
HTN	53%	53%	49%	58%	0.56	0.76
OSA	21%	5%	15%	46%	18.37	<0.001
T2D	21%	11%	13%	42%	13.26	0.001
MAP	95.4 (1.1)	94.2 (2.2)	95.0 (1.8)	97.2 (1.9)	0.55	0.58

*MCI, mild cognitive impairment; BMI, body mass index; T2D, type 2 diabetes; HTN, hypertension; HLD, hyperlipidemia; OSA, obstructive sleep apnea; GERD, gastroesophageal reflux disease; MAP, mean arterial pressure; NW, normal weight; OW, overweight; OB, obese. Values reported as percentages for categorical variables and mean (SE) for continuous variables. The statistic is chi-square test of independence (2 degrees of freedom) for categorical variables and an ANOVA *F*-statistic for continuous variables.*

The demographics of subjects with and without T2D and OSA are presented in **Table 3**. Age and MCI-SV were similar between groups although education and MMSE score were lower in subjects with T2D. The NPI-Q mean total score was significantly higher in subjects with T2D and OSA. Further, the prevalence of moderate level NPI-Q symptoms differed based on the presence of T2D and OSA. Depression scores measured by the GDS were also significantly higher for T2D subjects. There was no difference in age, education, MMSE, MCI-SV, or GDS across OSA groups.

**Table 3:** Demographic, cognitive, and behavioral measures of T2D and OSA groups.

Characteristic	T2D	No-T2D	Statistic		OSA	No–OSA	Statistic	
	*N* = 23	*N* = 88	X^2^/*F*	*p*-value	*N* = 23	*N* = 88	X^2^ or *F*	*p*-value
Age (years)	73.0 (1.25)	74.6 (0.87)	1.06	0.29	72.7 (1.38)	74.7 (0.86)	1.07	0.287
Female, *n* (%)	12 (52.2)	40 (45.5)	0.33	0.57	7 (30.4)	45 (51.1)	3.14	0.076
Education (years)	13.3 (0.77)	14.9 (0.33)	2.01	0.047	15.0 (0.86)	14.4 (0.33)	0.64	0.450
BMI (kg/m^2^)	29.9 (1.08)	26.7 (0.47)	3.00	0.003	30.65 (0.92)	26.52 (0.48)	3.95	<0.001
MMSE	25.8 (0.36)	26.7 (0.19)	2.13	0.035	26.74 (0.37)	26.40 (0.19)	0.83	0.411
MCI-severity	-1.04 (0.15)	-0.86 (0.06)	1.21	0.230	-0.73 (0.18)	-0.93 (0.06)	1.27	0.208
NPI-Q score	7.63 (1.18)	4.48 (0.62)	2.38	0.019	7.75 (1.37)	4.59 (0.60)	2.23	0.028
≥1, *n* (%)	17 (89.5)	51 (76.1)	1.60	0.338	14 (87.5)	54 (77.1)	0.84	0.505
≥4, *n* (%)	14 (73.7)	27 (40.3)	6.61	0.010	11 (68.8)	30 (42.9)	3.50	0.061
GDS score	4.26 (0.87)	2.67 (0.26)	2.36	0.020	3.76 (0.85)	2.77 (0.25)	1.12	0.274
≥1, *n* (%)	18 (94.7)	71 (85.5)	1.18	0.453	18 (85.7)	71 (87.7)	0.056	0.812
≥4, *n* (%)	9 (47.4)	22 (26.5)	3.18	0.075	8 (38.1)	23 (28.4)	0.74	0.389

*BMI, body mass index; MMSE, Mini-Mental State Examination; MCI-SV, MCI severity; GDS, Geriatric Depression Scale; NPIQ, Neuropsychiatric Inventory Questionnaire; T2D, type 2 diabetes; OSA, obstructive sleep apnea. Results are presented as mean (SE) for continuous variables and *n* (%) for categorical variables. The statistic is chi-square test of independence (1 degree of freedom) for categorical variables and an independent samples *t*-test, *t*-statistic for continuous variables.*

### NPI-Q Clusters

**FIGURE 1:**
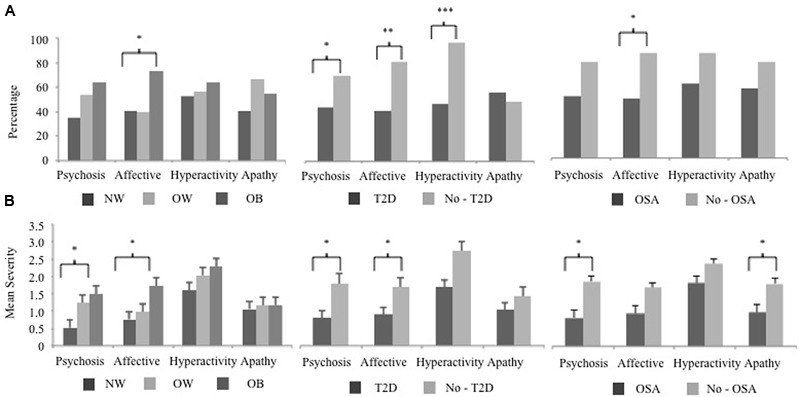
**The NPI-Q cluster frequency and severity of BMI, T2D, and OSA MCI subject groups.** The 12 NPI-Q symptoms domains are clustered into four groups of, Hyperactivity (agitation, disinhibition, irritability, motor disturbances, and euphoria), Apathy (apathy, appetite), Affective (depression, anxiety) and Psychosis (delusions, hallucinations, night-time behaviors). **(A)** The frequency of each NPI-Q cluster is plotted for BMI, T2D, and OSA groups. Cluster frequency statistics were conducted using the chi-square test of independence (2 degrees of freedom for BMI and 1 degree of freedom for T2D and OSA). **(B)** The mean (SE) severity of NPI-Q clusters for BMI, T2D, and OSA groups. Mean differences in cluster severity were compared using the analysis of variance (ANOVA) model for BMI and a Student’s *t*-test for T2D and OSA. Significant associations are marked as follow, ^∗^*p* < 0.05, ^∗∗^*p* < 0.01, ^∗∗∗^*p* < 0.001. NPI-Q, Neuropsychiatric Inventory Questionnaire; MCI, mild cognitive impairment; BMI, body mass index; T2D, type 2 diabetes; OSA, obstructive sleep apnea.

The prevalence and severity of specific NPI-Q clusters differed with respect to MCI subjects who were obese, and had T2D or OSA. The Hyperactivity cluster was the most frequent with 56% of subjects having at least one symptom. **Figure 1A** shows the frequency of each symptom cluster across groups. Affective symptoms significantly differed between OB and NW groups ($X_{2}^{2}$ = 6.76, *p* = 0.03). Subjects with sleep apnea also showed a significantly higher frequency of solely Affective symptoms ($X_{2}^{2}$ = 5.39, *p* = 0.02). Diabetic subjects had a significantly higher frequency across three clusters of, Affective ($X_{2}^{2}$ = 8.85, *p* = 0.003), Hyperactivity ($X_{2}^{2}$ = 14.19, *p* < 0.001) and Psychosis ($X_{2}^{2}$ = 3.74, *p* = 0.05) symptoms. Next, a posterior power analysis was conducted to measure the strength of the association between obesity, T2D, and OSA with each NPI-Q clusters. For our significant comparisons of OB and OSA with Affective symptoms had a power of 60 and 56% respectively. In addition, for each significant association of T2D, as the cluster significance decreased, the power of the association increased: Psychosis, power = 50%, Affective, power = 86%, and Hyperactivity, power = 98%.

The mean severity of NPI-Q clusters across groups is shown in **Figure 1B**. In OB subjects, Affective symptoms were also more severe (*F* = 3.30, *p* = 0.04) along with the Psychosis cluster (*F* = 4.55, *p* = 0.03). Subjects with OSA also had a higher severity of Psychosis symptoms (Student’s *t* = 2.50, *p* = 0.02) as well as Apathy (Student’s *t* = 2.17, *p* = 0.03) compared to those without a sleep disorder. Two NPS clusters were more severe in diabetic subjects were, Affective (Student’s *t* = 2.11, *p* = 0.04), and Psychosis (Student’s *t* = 2.52, *p* = 0.02) clusters, while Apathy and Hyperactivity were unrelated to this condition.

## Discussion

In this study, we assessed the relationship of weight with specific neuropsychiatric symptoms and the severity of cognitive impairment in mild cognitive impairment subjects. Our hypothesis was supported, in part, in that the frequency and severity of affective symptoms were significantly higher in obese subjects. To our knowledge, a direct examination of the relationship between body mass index and neuropsychiatric symptoms in mild cognitive impairment has not been reported, although the prevalence of each weight group in our sample is similar to national averages of overweight and obese individuals in the adult US population (Ogden et al., 2014). While there is a growing body of literature on the effects of obesity on behavioral symptoms and cognition we sought to include in our analysis obesity-related conditions which often occur co-morbidly (Kandimalla et al., 2016) and are believed to share neuropathological commonalities. Interestingly, HTN, HL, and GERD proved not to be significantly related to obesity in our sample; they were present in relatively equal proportions in all 3 BMI groups (although HL showed a definite trend toward increase). This likely speaks to the multifactorial nature of these conditions, such that the contribution of obesity is only one of several driving factors. Our results indicate that in MCI the combination of increased weight with T2D showed the greatest differences in behavioral disturbances in regards to total scores, symptom cluster frequency and severity as well as changes in global cognition.

In our sample of early stage MCI subjects, BMI and related health conditions demonstrated a significantly higher prevalence and severity of specific NPS. Previous studies have reported depression, anxiety and apathy symptoms as the most frequent NPS seen in MCI, among obese persons (Simon et al., 2006; Luppino et al., 2010) as well as in subjects with T2D (Katon et al., 2012) and OSA (Akmal et al., 2013). Our study supports these findings in that the Affective cluster (depression, anxiety) was more frequent in subjects with OB, T2D, and OSA compared to those that were NW/OW or without T2D and OSA. The Affective cluster was also rated with greater severity for OB and T2D subjects, which leads to our main finding that that there is a relationship between depression and anxiety and obesity in MCI. For all groups the Psychosis cluster had a significant difference in mean severity although it was only more prevalent in the T2D group. A possible explanation may be that despite delusions and hallucinations constituting the least frequent symptoms in MCI (Apostolova and Cummings, 2008; Geda et al., 2008), their presence in the early stage of cognitive impairment is perceived more severely by the informant. Further, the presence of night-time behaviors in this cluster were most likely the driving factor: nighttime behaviors were the most frequent and severe individual NPI-Q symptom present in 42% of subjects. The Apathy cluster did not differ in frequency in any of the group comparisons; however, it did have a higher severity only in OSA subjects. Higher apathy in relationship to daytime sleepiness has been shown in OSA subjects (Vernet et al., 2011), which may explain the heightened severity rating when present in this group. Thus, when assessing MCI subjects with behavioral disturbances, consideration should be given to higher BMI and BMI-related health conditions, specifically T2D and OSA, as possible contributors to the presentation of NPS. Future research will be necessary to determine whether lifestyle interventions and treatment of weight related disorders affect the persistence and severity of NPS over time.

The link between weight-related health conditions and NPS is not well-understood. As with similar findings between these health conditions and cognition, current research has begun to identify central inflammation as a possible mechanism. One theory postulates that weight gain modulates adipocyte function resulting in a higher secretion of pro-inflammatory markers that reach the brain and alter neuronal function, ultimately leading to alterations in neurocircuitry and neural plasticity. These changes affect brain regions such as the prefrontal cortex and cingulate gyrus, resulting in the presentation of NPSs (Castanon et al., 2014). Moreover, a recent animal study showed a possibly direct effect of obesity on dopamine receptor function resulting in depression-like behaviors and alterations in reward circuitry (Sharma et al., 2013). In MCI, obesity is considered to be a chronic condition yet the time course of the indirect changes described by the mechanism of central inflammation is unclear. Further research is needed to understand whether weight-related brain changes present in conjunction with the onset of cognitive impairment or whether they act separately to promote the presentation of NPSs.

In contrast to our hypothesis, MCI severity was not associated with BMI or T2D and OSA groups. One explanation may be that MCI is defined by cognitive impairment and represents a transitional state with a narrow range of deficits. There is a cut-off to the severity that reflects MCIs before one achieves psychometric criteria for dementia. Moreover our study subjects are diagnosed as MCI by a stringent criterion of -1.5 SD in at least one cognitive domain, which in other studies has been broader (e.g., -1.0 SD in 2 cognitive domains). This difference in criteria may provide a more uniform assessment of overall MCI severity. Another possibility is that an overall severity score is not a sufficiently nuanced measure of cognitive status. Diabetes and OSA show greater cognitive deficits in executive function than memory. It may be more effective to measure individual cognitive domain severity in order to detect differences in the effects of disorders such as T2D, OSA, and even OB. Finally, overall MCI severity may differentiate groups later in the disease course, which cannot be examined in a cross-sectional design. However, one research study showed that MCI subjects with at least one symptom on the NPI-Q or GDS, and lower initial cognitive status resulted in a more rapid development of dementia (Rosenberg et al., 2012a). In our T2D subjects, general cognition measured by the MMSE was significantly lower (*p* = 0.03) while NPI-Q and GDS total scores were nearly doubled compared to subjects without T2D. This may indicate that MCI subjects with T2D and NPS ≥ 4 are at an increased risk for conversion to dementia.

A surprising finding of this study was the lower mean age of overweight and obese MCI subjects at the time of diagnosis by nearly 7 years. Middle age obesity promotes a higher risk of conversion to AD (Gustafson, 2008). Since our data come from newly diagnosed MCI patients, this suggests that higher adiposity may cause an earlier emergence of cognitive impairment, likely through the burden of additional physiologic stressors. Further, an early onset of cognitive impairment in the obese may create a group of individuals susceptible to the onset of AD at an earlier age compared to those of normal weight. Despite many OB subjects having T2D and OSA, there was not a difference in the age at diagnosis based on these conditions. Isolating risk factors associated with weight may unmask features of the underlying pathological changes associated with prodromal AD.

### Limitations

There are some limitations that must be taken into account in interpreting our results. First, this study is cross sectional and therefore does not assess cognitive and NPS status over time in relationship to BMI groups. For the same reason, it also cannot evaluate the direction of the association or capture the initiation and persistence of NPS. Second, due to the sample being a specialty referral clinical, genetic testing was not routinely done to establish apolipoprotein allele status, chronicity of overweight and obesity, or effectively capture socio economic status. In addition, our examination of NPI-Q symptomology was based on a score of ≥1, which is a very mild disturbance. However, recent studies have shown that even the measurement of the presence or absence of symptoms can predict disease progression (Rosenberg et al., 2012b). In this regard, it is notable that large differences were seen in the frequency and severity of NPI-Q clusters with respect to BMI-related disorders T2D and OSA.

Lastly, in our analysis we did not have adequate power to detect some of our associations between OB and OSA with affective symptoms. The results provided in this article allow for the generation of hypothesis for future work. This initial investigation provides new information about possible co-morbidities in MCI that can be replicated in a larger sample such as the Alzheimer’s Disease Neuroimaging Initiative (ADNI) (Petersen et al., 2010). The current focus of this research group is to further analyze the relationships of NPS and OB in MCI using a more rigorous epidemiologic approach with subjects from the ADNI dataset. Future studies will focus on longitudinal follow-up to examine whether there is a relationship between weight, NPS prevalence and MCI severity at later stages of MCI and early AD, and also to establish whether a higher BMI produces a greater incidence of NPS over time.

## Conclusion

This study demonstrates that within mild cognitive impairment, body mass index and related disorders, type-2-diabetes mellitus and obstructive sleep apnea, showed a higher rate of psychopathologic changes, most particularly in the Affective, Hyperactivity and Psychosis clusters. Further, increased late life adiposity, which represented over 65% of subjects, was associated with a lower mean age at the onset of cognitive symptoms. Future research should focus on better understanding the intersection of neuropsychiatric symptoms and obesity in mild cognitive impairment, as well as the combined effect of these disorders and body mass index-related disorders on the brain and clinical progression of mild cognitive impairment. In clinical settings diabetic patients with mild cognitive impairment should be monitored for behavioral changes.

## Author Contributions

AS: the corresponding author is responsible for the design of the experiment, as well as collecting and analyzing the research data. She also contributed as the primary author of the article. Dr. AB: is the lead neurologist that administered care for all of the patients in the study. She confirmed the diagnosis of MCI and contributed to the clinical aspects of the methodology and development of the discussion outline. Dr. DT: contributed by designing the statistical analysis procedures. He also contributed to the writing of the methods section and overall editing of the manuscript.

## Conflict of Interest Statement

The authors declare that the research was conducted in the absence of any commercial or financial relationships that could be construed as a potential conflict of interest.

## References

[B1] Aalten P. de Vugt M. E. Jaspers N. Jolles J. Verhey F. R. J. (2005). The course of neuropsychiatric symptoms in dementia. Part II: relationships among behavioural sub-syndromes and the influence of clinical variables. *Int. J. Geriatr. Psychiatry* 20 531–536. 10.1002/gps.1317 15920706

[B2] Aalten P. Verhey F. Boziki M. Bullock R. Byrne E. J. Camus V. (2007). Neuropsychiatric syndromes in dementia. Results from the European alzheimer disease consortium: part I. *Dement. Geriatr. Cogn. Disord.* 24 457–463. 10.1159/000110738 17986816

[B3] Akmal M. K. Ezat M. Raafat O. Hamed H. Bediwy A. (2013). Association of depression, anxiety, and impairment in executive functions in patients with obstructive sleep apnea. *Middle East Curr. Psychiatry.* 20 30–34. 10.1097/01.XME.0000422808.09000.59

[B4] Apostolova L. G. Cummings J. L. (2008). Neuropsychiatric manifestations in mild cognitive impairment: a systematic review of the literature. *Dement. Geriatr. Cogn. Disord.* 25 115–126. 10.1159/000112509 18087152

[B5] Atkinson R. Shiffrin R. (1971). The control of short-term memory. *Sci. Am.* 225 82–90. 10.1038/scientificamerican0871-82 5089457

[B6] Beydoun M. Beydoun H. Wang Y. (2008). Obesity and central obesity as risk factors for incident dementia and its subtypes: a systematic review and meta-analysis. *Obes. Rev.* 9 204–218. 10.1111/j.1467-789X.2008.00473.x 18331422 PMC4887143

[B7] Borkowski J. Benton A. Spreen O. (1967). Word fluency and brain damage. *Neuropsychologia* 5 135–140. 10.1016/0028-3932(67)90015-2

[B8] Castanon N. Lasselin J. Capuron L. (2014). Neuropsychiatric comorbidity in obesity: role of inflammatory processes. *Front. Endocrinol.* 5:74 10.3389/fendo.2014.00074 PMC4030152 24860551

[B9] Flegal K. M. Carroll M. D. Ogden C. L. Curtin L. R. (2010). Prevalence and trends in obesity among US adults, 1999-2008. *JAMA* 303 235–241. 10.1001/jama.2009.2014 20071471

[B10] Folstein M. Folstein S. McHugh P. (1975). Mini-mental state: a practical method for grading the cognitive state of patients for the clinician. *J. Psychiatr. Res.* 12 189–198. 10.1016/0022-3956(75)90026-6 1202204

[B11] Geda Y. Roberts R. Knopman D. S. Petersen R. C. Christianson T. J. Pankratz V. S. (2008). Prevalence of neuropsychiatric symptoms in mild cognitive impairment and normal cognitive aging: population-based study. *Arch. Gen. Psychiatry* 65 1193–1198. 10.1001/archpsyc.65.10.1193 18838636 PMC2575648

[B12] Gorska-Ciebiada M. Saryusz-Wolska M. Ciebiada M. Loba J. (2014). Mild cognitive impairment and depressive symptoms in elderly patients with diabetes: prevalence, risk factors, and comorbidity. *J. Diabetes Res.* 2014:179648 10.1155/2014/179648 PMC4241577 25431771

[B13] Gustafson D. (2006). Adiposity indices and dementia. *Lancet Neurol.* 5 713–720. 10.1016/S1474-4422(06)70526-9 16857578

[B14] Gustafson D. A. (2008). Life course of adiposity and dementia. *Eur. J. Pharmacol.* 585 163–175. 10.1016/j.ejphar.2008.01.052 18423446

[B15] Han T. Tajar A. Lean M. (2011). Obesity and weight management in the elderly. *Br. Med. Bull.* 97 169–196. 10.1093/bmb/ldr002 21325341

[B16] Herrman N. Mittmann N. Silver I. L. Shulman K. I. Busto U. A. Shear N. H. (1996). A validation study of the Geriatric Depression Scale short form. *Int. J. Geriatr. Psychiatry* 11 457–460. 10.1002/(SICI)1099-1166(199605)11:5<457::AID-GPS325>3.0.CO;2-2

[B17] Ismail Z. Smith E. E. Geda Y. Sultzer D. Brodaty H. Smith G. (2016). Neuropsychiatric symptoms as early manifestations of emergent dementia: provisional diagnostic criteria for mild behavioral impairment. *Alzheimers Dement.* 12 195–202. 10.1016/j.jalz.2015.05.017 26096665 PMC4684483

[B18] Kandimalla R. Thirumala V. Reddy P. H. (2016). Is Alzheimer’s disease a Type 3 Diabetes? A critical appraisal. *Biochim. Biophys. Acta Mol. Basis Dis.* 10.1016/j.bbadis.2016.08.018 [Epub ahead of print]. PMC5344773 27567931

[B19] Kaplan E. Goodglass H. Weintraub S. (1983). *The Boston Naming Test* 2nd Edn. Philadelphia, PA: Lea & Febiger.

[B20] Katon W. Lyles C. Parker M. M. Karter A. J. Huang E. S. Whitmer R. A. (2012). Association of depression with increased risk of dementia in patients with type 2 diabetes: the diabetes and aging study. *Arch. Gen. Psychiatry* 69 410–417. 10.1001/archgenpsychiatry.2011.154 22147809 PMC3558525

[B21] Kaufer D. I. Cummings J. L. Ketchel P. Smith V. MacMillan A. Shelley T. (2000). Validation of the NPI-Q, a brief clinical form of the neuropsychiatric inventory. *J. Neuropsychiatry Clin. Neurosci.* 12 233–239. 10.1176/jnp.12.2.233 11001602

[B22] Li J. Wang Y. J. Zhang M. Xu Z. Q. Gao C. Y. Fang C. Q. (2011). Vascular risk factors promote conversion from mild cognitive impairment to Alzheimer disease. *Neurology* 76 1485–1491. 10.1212/WNL.0b013e318217e7a4 21490316

[B23] Luppino F. de Wit L. M. Bouvy P. F. Stijnen T. Cuijpers P. Penninx B. W. (2010). Overweight, obesity, and depression: a systematic review and meta-analysis of longitudinal studies. *Arch. Gen. Psychiatry* 67 220–229. 10.1001/archgenpsychiatry.2010.2 20194822

[B24] Lyketsos C. Lopez O. Jones B. Fitzpatrick A. L. Breitner J. (2002). DeKosky SPrevalence of neuropsychiatric symptoms in dementia and mild cognitive impairment: results from the cardiovascular health study. *JAMA* 288 1475–1483. 10.1001/jama.288.12.1475 12243634

[B25] Misiak B. Leszek J. Kiejna A. (2012). Metabolic syndrome, mild cognitive impairment and Alzheimer’s disease–the emerging role of systemic low-grade inflammation and adiposity. *Brain Res. Bull.* 89 144–149. 10.1016/j.brainresbull.2012.08.003 22921944

[B26] Moms J. Heyman A. Mohs R. Hughes J. P. van Belle G. Fillenbaum G. (1989). The consortium to establish a registry for Alzheimer’s disease (CERAD). Part I. Clinical and neuropsychological assessment of Alzheimer’s disease. *Neurology* 39 1159–1165. 10.1212/WNL.39.9.1159 2771064

[B27] Ogden C. L. Carroll M. D. Kit B. K. Flegal K. M. (2014). Prevalence of childhood and adult obesity in the United States, 2011-2012. *JAMA* 311 806–814. 10.1001/jama.2014.732 24570244 PMC4770258

[B28] Palmer K. Berger A. Monastero R. Winblad B. Bäckman L. Fratiglioni L. (2007). Predictors of progression from mild cognitive impairment to Alzheimer disease. *Neurology* 68 1596–1602. 10.1212/01.wnl.0000260968.92345.3f 17485646

[B29] Petersen R. Smith G. Waring S. C. Ivnik R. J. Tangalos E. G. Kokmen E. (1999). Mild cognitive impairment: clinical characterization and outcome. *Arch. Neurol.* 56 303–308. 10.1001/archneur.56.3.303 10190820

[B30] Petersen R. C. Aisen P. S. Beckett L. A. Donohue M. C. Gamst A. C. Harvey D. J. (2010). Alzheimer’s disease Neuroimaging Initiative (ADNI) clinical characterization. *Neurology* 74 201–209. 10.1212/WNL.0b013e3181cb3e25 20042704 PMC2809036

[B31] Rosen W. Mohs R. Davis K. A. (1984). New rating scale for Alzheimer’s disease. *Am. J. Psychiatry* 141 1356–1364. 10.1176/ajp.141.11.1356 6496779

[B32] Rosenberg P. B. Mielke M. M. Appleby B. S. Oh E. S. Geda Y. E. Lyketsos C. G. (2012a). The association of neuropsychiatric symptoms in MCI with incident dementia and Alzheimer disease. *Am. J. Geriatr. Psychiatry* 10.1097/JGP.0b013e318252e41a [Epub ahead of print] PMC3428504 23567400

[B33] Rosenberg P. B. Mielke M. M. Appleby B. Oh E. Leoutsakos J. Lyketsos C. G. (2012b). Neuropsychiatric symptoms in MCI subtypes: the importance of executive dysfunction. *Int. J. Geriatr. Psychiatry* 26 364–372. 10.1002/gps.2535.Neuropsychiatric PMC3204866 20845402

[B34] Sharma S. Fernandes M. Fulton S. (2013). Adaptations in brain reward circuitry underlie palatable food cravings and anxiety induced by high-fat diet withdrawal. *Int. J. Obes.* 37 1183–1191. 10.1038/ijo.2012.197 23229740

[B35] Simon G. E. Von Korff M. Saunders K. Miglioretti D. L. Crane P. K. van Belle G. (2006). Association between obesity and psychiatric disorders in the US adult population. *Arch. Gen. Psychiatry* 63 824–830. 10.1001/archpsyc.63.7.824 16818872 PMC1913935

[B36] Steinberg M. Hess K. Corcoran C. Mielke M. M. Norton M. Breitner J. (2014). Vascular risk factors and neuropsychiatric symptoms in Alzheimer’s disease: the Cache County Study. *Int. J. Geriatr. Psychiatry* 29 153–159. 10.1002/gps.3980 23681754 PMC3883945

[B37] Stroop J. (1935). Studies of interference in serial verbal reactions. *J. Exp. Psychol.* 18 643–662. 10.1037/h0054651

[B38] Teng E. Chui H. (1987). The modified mini-mental state (3MS) examination. *J. Clin. Psychiatry* 48 314–318. 3611032

[B39] Teng W. Lu P. H. Cummings J. L. (2007). Neuropsychiatric symptoms are associated with progression from mild cognitive impairment to Alzheimer’s disease. *Dement. Geriatr. Cogn. Disord.* 24 253–259. 10.1159/000107100 17700021

[B40] Tombaugh T. (2004). Trail making test A and B: normative data stratified by age and education. *Arch. Clin. Neuropsychol.* 10 203–214. 10.1016/S0887-6177(03)00039-8 15010086

[B41] Vernet C. Redolfi S. Attali V. Konofal E. Brion A. Frija-Orvoen E. (2011). Residual sleepiness in obstructive sleep apnoea: phenotype and related symptoms. *Eur. Respir. J.* 38 98–105. 10.1183/09031936.00040410 21406511

[B42] Whitmer R. Gustafson D. R. Barrett-Connor E. Haan M. N. Gunderson E. P. Yaffe K. (2008). Central obesity and increased risk of dementia more than three decades later. *Neurology* 71 1057–1064. 10.1212/01.wnl.0000306313.89165.ef 18367704

[B43] Zhao Q.-F. Tan L. Wang H.-F. Jiang T. Tan M. S. Tan L. (2016). The prevalence of neuropsychiatric symptoms in Alzheimer’s disease: systematic review and meta-analysis. *J. Affect. Disord.* 190 264–271. 10.1016/j.jad.2015.09.069 26540080

