# Corrigendum: Obesity and Co-morbid Conditions Are Associated with Specific Neuropsychiatric Symptoms in Mild Cognitive Impairment

**DOI:** 10.3389/fnagi.2017.00325

**Published:** 2017-10-09

**Authors:** Ashley H. Sanderlin, David Todem, Andrea C. Bozoki

**Affiliations:** ^1^Neuroscience Program, Michigan State University, East Lansing, MI, United States; ^2^Division of Biostatistics, Department of Epidemiology and Biostatistics, Michigan State University, East Lansing, MI, United States; ^3^Department of Neurology and Ophthalmology, Michigan State University, East Lansing, MI, United States

**Keywords:** mild cognitive impairment, behavioral symptoms, obesity, Alzheimer's disease, Type 2 diabetes

In the original article, there was a mistake in Figure 1 as published. The color coded label for subject groups was switched for T2D and OSA groups. The light bars should be for the T2D and the OSA groups and the dark bars should be for the no-T2D and no-OSA groups. The legend and explanations of the results are all stated correctly to reflect the result as it should have appeared. The corrected Figure 1 appears below.

**Figure 1:**
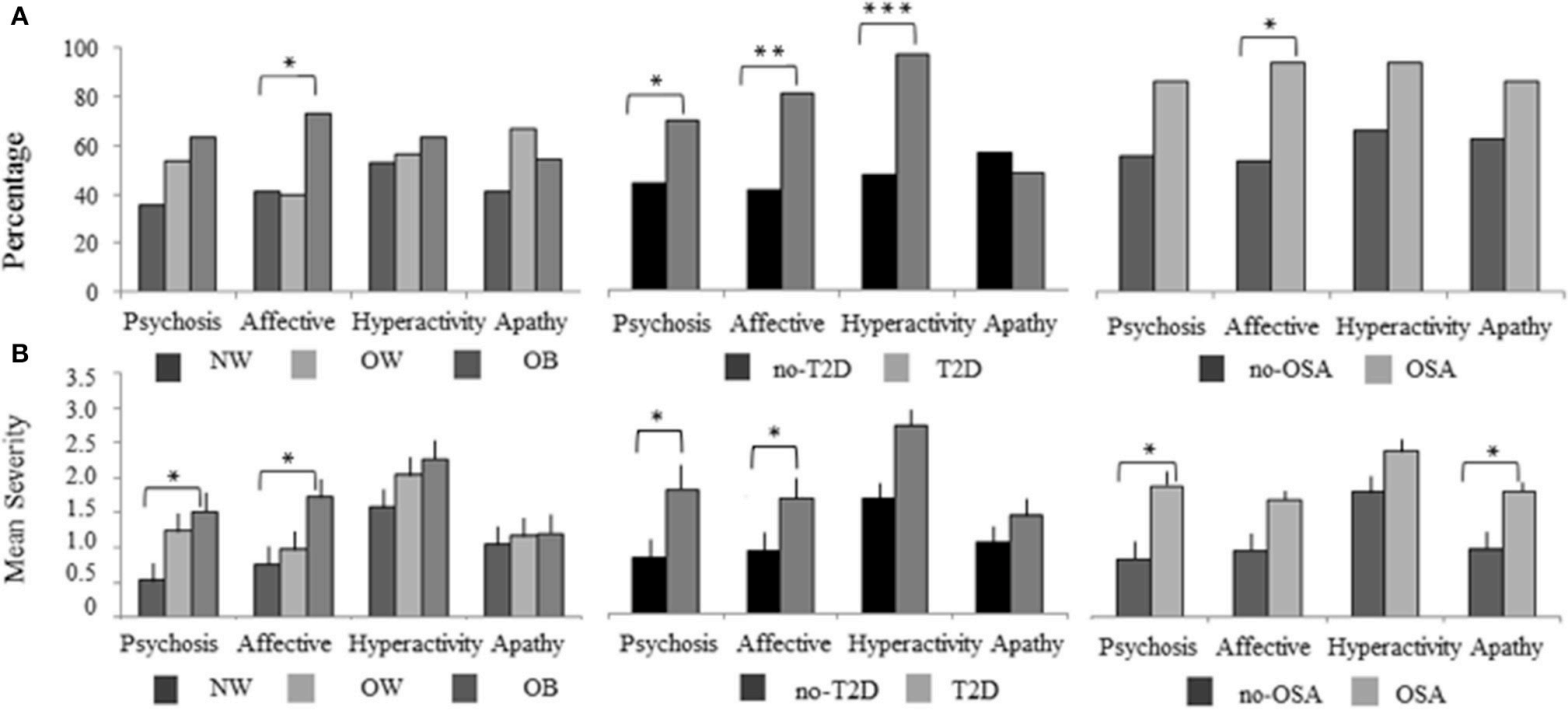
The NPI-Q cluster frequency and severity of BMI, T2D, and OSA MCI subject groups. The 12 NPI-Q symptoms domains are clustered into four groups of, Hyperactivity (agitation, disinhibition, irritability, motor disturbances, and euphoria), Apathy (apathy, appetite), Affective (depression, anxiety) and Psychosis (delusions, hallucinations, night-time behaviors). **(A)** The frequency of each NPI-Q cluster is plotted for BMI, T2D, and OSA groups. Cluster frequency statistics were conducted using the chi-square test of independence (2 degrees of freedom for BMI and 1 degree of freedom for T2D and OSA). **(B)** The mean (SE) severity of NPI-Q clusters for BMI, T2D, and OSA groups. Mean differences in cluster severity were compared using the analysis of variance (ANOVA) model for BMI and a Student's *t*-test for T2D and OSA. Significant associations are marked as follow, ^*^p < 0.05, ^**^p < 0.01, ^***^p < 0.001. NPI-Q, Neuropsychiatric Inventory Questionnaire; MCI, mild cognitive impairment; BMI, body mass index; T2D, type 2 diabetes; OSA, obstructive sleep apnea.

In the original article, there was a mistake in Table 3 as published. The statistic column indicated the measures were χ^2^ or *F* when it should have said χ^2^ or *t*. The legend description and statistics for Table 3 are all correct. The headings for the columns followed the format of Tables 1–2 but should have a *t* not *F* written to reflect the test conducted. The corrected Table 3 appears below.

**Table 3:** Demographic, cognitive, and behavioral measures of T2D and OSA groups.

**Characteristic**	**T2D *N* = 23**	**No-T2D *N* = 88**	**Statistic χ^2^ or *t***	***p*-value**	**OSA *N* = 23**	**No-OSA *N* = 88**	**Statistic χ^2^ or *t***	***p*-value**
Age (years)	73.0 (1.25)	74.6 (0.87)	1.06	0.29	72.7 (1.38)	74.7 (0.86)	1.07	0.287
Female, *n* (%)	12 (52.2)	40 (45.5)	0.33	0.57	7 (30.4)	45 (51.1)	3.14	0.076
Education (years)	13.3 (0.77)	14.9 (0.33)	2.01	0.047	15.0 (0.86)	14.4 (0.33)	0.64	0.450
BMI (kg/m2)	29.9 (1.08)	26.7 (0.47)	3.00	0.003	30.65 (0.92)	26.52 (0.48)	3.95	<0.001
MMSE	25.8 (0.36)	26.7 (0.19)	2.13	0.035	26.74 (0.37)	26.40 (0.19)	0.83	0.411
MCI-severity	−1.04 (0.15)	−0.86 (0.06)	1.21	0.230	−0.73 (0.18)	−0.93 (0.06)	1.27	0.208
NPI-Q score	7.63 (1.18)	4.48 (0.62)	2.38	0.019	7.75 (1.37)	4.59 (0.60)	2.23	0.028
≥1, *n* (%)	17 (89.5)	51 (76.1)	1.60	0.338	14 (87.5)	54 (77.1)	0.84	0.505
≥4, *n* (%)	14 (73.7)	27 (40.3)	6.61	0.010	11 (68.8)	30 (42.9)	3.50	0.061
GDS score	4.26 (0.87)	2.67 (0.26)	2.36	0.020	3.76 (0.85)	2.77 (0.25)	1.12	0.274
≥1, *n* (%)	18 (94.7)	71 (85.5)	1.18	0.453	18 (85.7)	71 (87.7)	0.056	0.812
≥4, *n* (%)	9 (47.4)	22 (26.5)	3.18	0.075	8 (38.1)	23 (28.4)	0.74	0.389

In the original article, there was an error. In the discussion the word BMI was used where the word obesity should have been placed when discussing the MCI severity results. There was a difference across BMI groups with a *p*-value of 0.02 but this significance was not associated with obesity which should have been stated. A correction has been made to the Discussion section, paragraph 4:

In contrast to our hypothesis, MCI severity was not associated with obesity or T2D and OSA groups. The severity of cognitive symptoms was greatest for normal weight subjects although there was no difference in MMSE scores. One explanation may be that MCI is defined by cognitive impairment and represents a transitional state with a narrow range of deficits. There is a cut-off to the severity that reflects mild cognitive impairments before one achieves psychometric criteria for dementia. Moreover our study subjects are diagnosed as MCI by a stringent criterion of −1.5 SD in at least one cognitive domain, which in other studies has been broader, (e.g., −1.0 SD in 2 cognitive domains). This difference in criteria may provide a more uniform assessment of overall MCI severity. Another possibility is that an overall severity score is not a sufficiently nuanced measure of cognitive status. Diabetes and OSA show greater cognitive deficits in executive function than memory. It may be more effective to measure individual cognitive domain severity in order to detect differences in the effects of disorders such as T2D, OSA and even OB. Finally, MCI severity may differentiate groups later in the disease course, which cannot be examined in a cross-sectional design. However, one research study showed that MCI subjects with at least one symptom on the NPI-Q or GDS, and lower initial cognitive status resulted in a more rapid development of dementia (Rosenberg et al., 2013). In our T2D subjects, general cognition measured by the MMSE was significantly lower (*p* = 0.03) while NPI-Q and GDS total scores were nearly doubled compared to subjects without T2D. This may indicate that MCI subjects with T2D and NPS ≥ 4 are at an increased risk for conversion to dementia.

The authors apologize for these errors and state that this does not change the scientific conclusions of the article in any way.

## Conflict of interest statement

The authors declare that the research was conducted in the absence of any commercial or financial relationships that could be construed as a potential conflict of interest.
